# Dr. Matthew Joseph O’Connell

**Published:** 1914-10

**Authors:** 


Dr. Matthew Joseph O'Connell, Niagara, 1890, superintendent of a sanitarium for the treatment of alcohol and drug addictions, died in Buffalo, August 4, of acute gastritis, aged 52.

